# Evaluation of Localized Spallation of TBCs via a Combination of Conjugate Heat Transfer Numerical and Experimental Analysis

**DOI:** 10.3390/ma17133102

**Published:** 2024-06-25

**Authors:** Fan Sun, Peng Jiang, Jianpu Zhang, Yiwen Chen, Dingjun Li

**Affiliations:** 1 State Key Lab for Strength and Vibration of Mechanical Structures, Department of Engineering Mechanics, Xi’an Jiaotong University, Xi’an 710049, China; sf2038945624@163.com (F.S.); chenyiwenac@163.com (Y.C.); 2 State Key Laboratory of Clean and Efficient Turbomachinery Power Equipment, Dongfang Steam Turbine Co., Ltd., Deyang 618000, China; zhang695975990@126.com (J.Z.); lidingjun@dongfang.com (D.L.)

**Keywords:** thermal barrier coating, localized spallation, temperature distribution, critical spallation width, over-temperature damage

## Abstract

To fully realize the potential application of spalled thermal barrier coating systems (TBCs) in gas turbine blades, it is essential to evaluate the service behavior of TBCs and the critical spallation size for safety servicing. For this purpose, the evaluation of the localized spallation of TBCs under high-temperature gas was investigated experimentally and numerically. Thermal insulation experiments and a conjugate heat transfer numerical algorithm were used to clarify the over-temperature phenomenon, temperature distributions, the relevant flow characteristics of the high-temperature gas in the localized spallation region of TBCs, and the influencing mechanisms that consider the spallation width were identified. The results suggested that when the spallation width was less than 10 μm, the temperature in the TBCs did not change due to the weak impression of gas. When the spallation width exceeded the security coefficient of about 3 mm, the TBCs were difficult to service safely due to the impact of high-temperature gas. Furthermore, the concept of an over-temperature coefficient was proposed to describe the over-temperature damage and a nonlinear fitting equation was obtained to reveal and predict the evolution of the over-temperature coefficient. The over-temperature coefficient may serve as a valuable metric in determining the performance degradation of TBCs.

## 1. Introduction

Thermal barrier coating systems (TBCs) have been widely used to improve the high-temperature durability and efficiency of superalloy blades in gas turbines [1,2,3,4,5,6]. However, the interfacial damage and surface damage of TBCs caused by thermal mismatch stress, sintering, thermally grown oxide (TGO) growth, particle erosion, and other factors inevitably occur during the service. These damages can lead to different degrees of impact on performance degradation, such as a decline in thermal insulation capability and structural integrity [7,8,9,10,11,12,13,14,15]. Moreover, a few adjacent micro-cracks gradually coalesce into a large crack exhibiting different sizes in the localized spallation, accelerating the premature failure of TBCs to varying degrees. To assess the extent of damage and enhance the full potential of the damaged TBCs, it is essential to evaluate the sustainability of damaged TBCs [16].

Issues on the evaluation of interfacial-damaged TBCs have been investigated extensively. Yanar et al. [17] pointed out that TBCs defects, transient oxides, and surface defects are the key factors causing TBCs interfacial damage. Mahfouz and Zhou et al. [18,19] conducted burner rig experiments and found that higher local temperatures and higher cooling rates have a significant effect on the increased interfacial damage rate. Beck and Trunova et al. [20,21] proposed a TBCs lifetime prediction method based on the accumulated interfacial crack length. Liu et al. [22] pointed out that the TBC life is directly correlated to the accumulative stress close to the top coat (TC)–bond coat (BC) interface. Then, Jiang et al. [8] developed a non-destructive photoluminescence piezo-spectroscopy (PLPS) method to measure the accumulative stress near the TBCs interface and obtain the relationship between the stress and the coating’s remaining life after thermal cycling. Nordhorn et al. [23] developed a numerical lifetime model based on the probabilistic fracture mechanical analyses and summarized statistical distributions of TBCs lifetime as a function of temperature.

**Figure 1:**
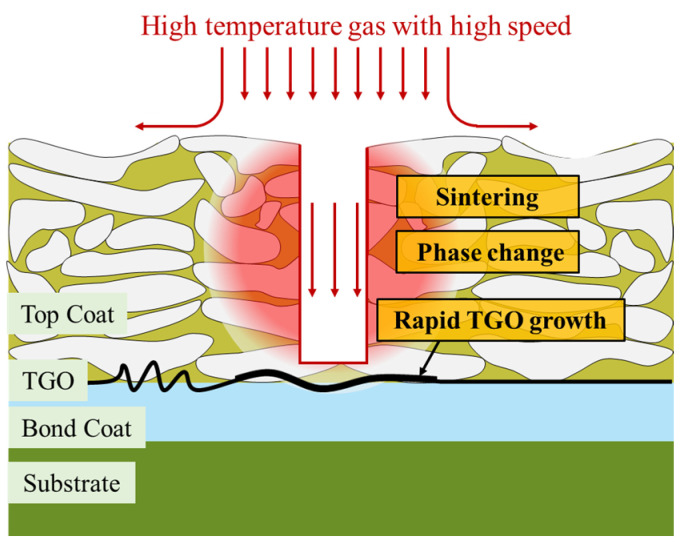
Schematic diagram of over-temperature damage in localized spallation region of TBCs.

Issues on the evaluation of surface-damaged TBCs are still controversial, with localized spallation being one of the most common modes, especially on the leading edge of blades (Figure 1). The localized spallation behavior may lead to the direct exposure of the damaged region to the high-temperature gas, resulting in the appearance of complicated gas flow characteristics and unpredictable temperature distributions in the spallation region. As the size of the spallation increases, the TBCs lose their protective function partially, and the temperature value in the affected region rises sharply above the normal value. This over-temperature phenomenon would accelerate sintering, phase changes, rapid TGO growth, and subsequent large-scale spallation and delamination, which leads to the premature failure of TBCs and even the cracking of the underlying blades. Researchers believe that there exists a critical size to evaluate the degree of localized spallation, above which the TBCs fail and below which it is regarded as sustainable, such as 5% [24], 15% [25], 25% [26,27], and so on. Unfortunately, up to now, such a critical size has not been unanimously adopted due to the lack of solid support. Therefore, understanding the temperature distribution in the localized spallation region of TBCs is crucial for determining the remaining life and availability of TBCs with localized spallation.

In this work, the over-temperature evaluation and the critical spallation size of TBCs are investigated experimentally and numerically. This paper is organized as follows: In Section 2, the TBC samples with different spallation widths are prepared, and thermal insulation tests are conducted to measure the temperature values of the spallation region. In Section 3, a conjugate heat transfer numerical analysis model of localized spallation on TBCs is presented to obtain the temperature distributions and relevant flow characteristics close to the spallation region, and the influencing mechanisms considering the spallation width are given. In Section 4, the numerical results and experimental data are analyzed. A dimensionless parameter, the over-temperature coefficient, is proposed to evaluate the over-temperature damage for the TBCs, and the variation law of the coefficient is also provided. Additionally, two critical widths for the damage evaluation of localized spallation on TBCs are identified using the validated numerical results. Finally, concluding remarks are drawn in Section 5.

## 2. Experimental Setup

To comprehend the effect of localized spallation on temperature distribution, TBC samples with different spallation widths were prepared, and thermal insulation experiments were conducted to measure temperatures at key locations.

### 2.1. Preparation of TBC Samples with Different Spallation Widths

**Figure 2:**
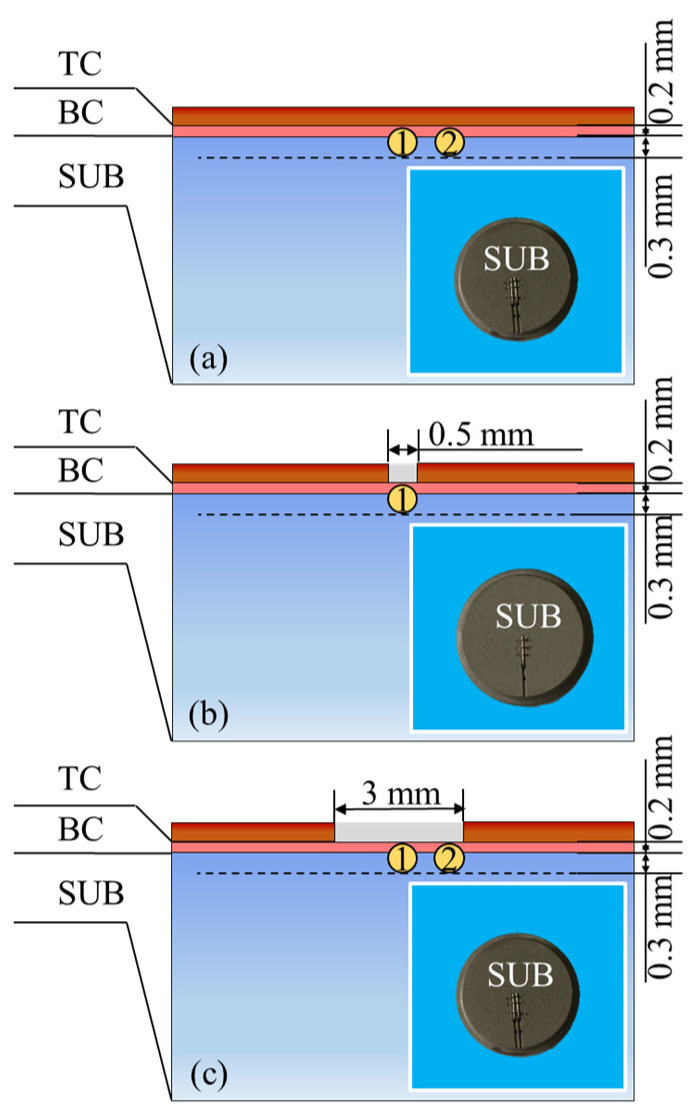
Thermocouples in TBC samples with spallation widths of (**a**) 0 mm, (**b**) 0.5 mm, and (**c**) 3 mm.

The substrate (SUB) layer of TBC samples was a cylinder made of nickel-based superalloy IN738, with a dimension of Φ 25 mm × 3 mm. The BC layer, 0.2 mm in thickness, was sprayed by the atmosphere plasma-spray (APS) gun (F4MB, Oerlikon Metco, Winterthur, Switzerland) using the standard commercial NiCoCrAlY powder (Co211, Oerlikon Metco, Winterthur, Switzerland). Before the spraying of the BC layer, grooves were formed at the BC surface using the spark erosion method in location 1 and location 2, with a depth of 300 μm and a width of 500 μm. High-precision type K thermocouples with a diameter of 500 μm were then embedded in location 1 and location 2 to monitor temperature values during the thermal insulation experiments, as shown in Figure 2. Location 1 and location 2 represent the center and edge of the exposed BC surface, respectively. Afterward, the TC layer, with a thickness of 300 μm, was sprayed by the APS gun using the commercial ZrO_2_-8wt% Y_2_O_3_ powder (Metco 204B-NS, Oerlikon Metco, Winterthur, Switzerland).

**Figure 3:**
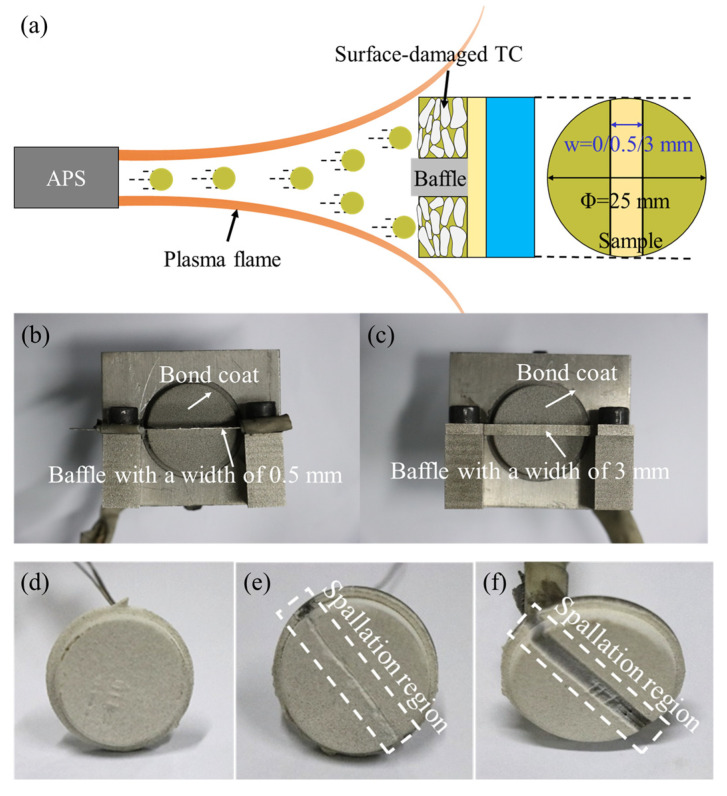
(**a**) Spraying process for TC layer, two baffles used for TBC samples with spallation widths of (**b**) 0.5 mm, and (**c**) 3 mm before the spraying of TC layer, and TBC samples with spallation widths of (**d**) 0 mm, (**e**) 0.5 mm, and (**f**) 3 mm.

**Figure 4:**
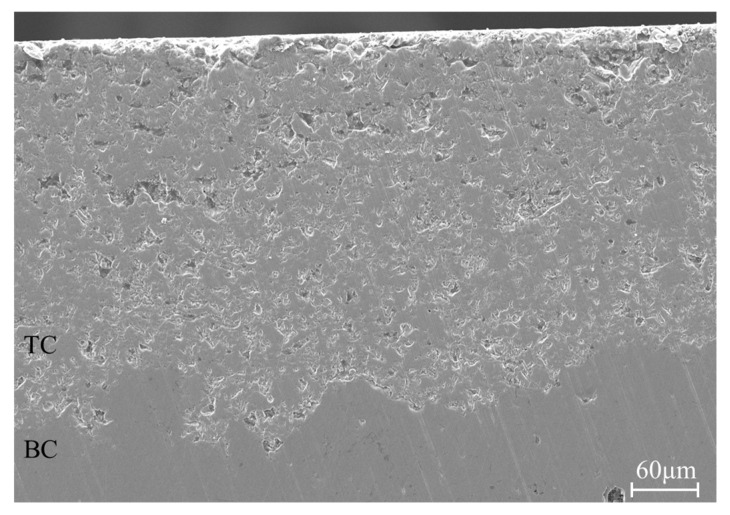
Microstructure of TC layer in APS TBCs.

Figure 3 displays the spraying process for the TC layer. To prepare the TC layer with spallation widths of 0 mm (i.e., the intact TBCs), 0.5 mm, and 3 mm, respectively, a clamp with baffles of varying widths was used during the TC spraying process (Figure 3b,c). Three samples with spallation widths of 0 mm (Figure 3d), 0.5 mm (Figure 3e), and 3 mm (Figure 3f) were prepared and the detailed thermal spray parameters for the BC layer and TC layer are listed in Table 1. During the spraying process, the plasma spray gun moved parallel to the surface of the sample, maintaining a fixed distance and angle between the sample and the spray gun and preparing the uniform TC and BC layers. The cross-sectional images of as-sprayed TBCs were obtained by scanning electron microscopy (SEM, SU3500, Hitachi, Tokyo, Japan), as shown in Figure 4.

### 2.2. Thermal Insulation Tests and Temperature Measurements

**Figure 5:**
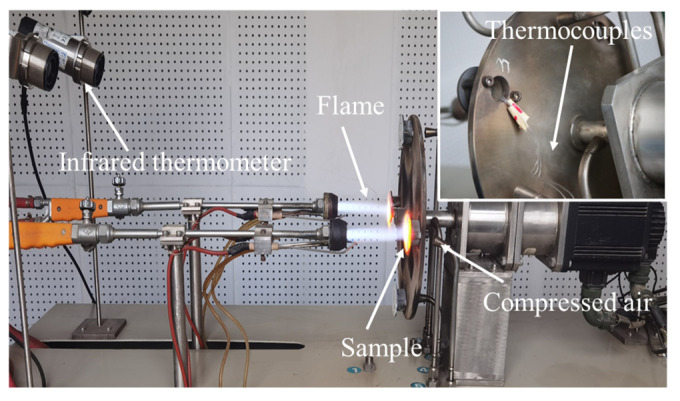
The gas burner rig test facility used for thermal insulation tests.

Thermal insulation tests were conducted on the localized spallation of TBCs to obtain the temperature values at the edge of the spallation region on the TC surface, as well as location 1 and location 2 on the exposed BC surface, thereby revealing the temperature variations influenced by the localized spallation. The tests were carried out using a gas burner rig test facility (Figure 5), where propane and oxygen were used as fuels. The TC surface was heated by gas flame, and the SUB back face was cooled with compressed air. A constant group of test parameters was selected: the distance between the flame nozzle and the sample surface was 100 mm, the flow rates of oxygen and propane were 7.5 L/min and 1.4 L/min, and the compressed air flow rate on the SUB back face was 25 L/min. 

The samples were heated for approximately 5 min and then cooled at ambient temperature. A commercial Single Lens Reflex (SLR) camera was used to capture the TC surface of TBC samples under a high-temperature flame. An infrared thermometer (AS872, Smart sensor, Hong Kong, China) was used to obtain the temperature values at the edge of the spallation region on the TC surface. The distance between the infrared thermometer and the TC surface was 10 cm, and the measurement area was circular with 2 mm in diameter. The wavelength and accuracy of the infrared thermometer were 8~14 μm and ±1.5%, respectively. The emissivity of the thermometer was set to 1. At the same time, the embedded thermocouples were used to measure the temperature values at location 1 and location 2.

## 3. Numerical Model

To understand the effect of spallation width on the temperature distribution in the localized spallation region of TBCs in-depth, and identify the over-temperature phenomenon and the relevant influencing mechanisms, the numerical model of the localized spallation of TBCs was constructed, and a numerical analysis was conducted. When the localized spallation region interacts with the high-temperature gas, complicated flow characteristics and unpredictable temperature distributions may occur in the localized spallation region (Figure 1), which is difficult to investigate by the fixed temperature boundary condition in most of the previous research [24,25]. Thus, to explore the accurate temperature distributions in the spallation region, a conjugate heat transfer (CHT) numerical model of the localized spallation of TBCs under high-temperature gas was developed.

### 3.1. Geometry and Meshed Model

**Figure 6:**
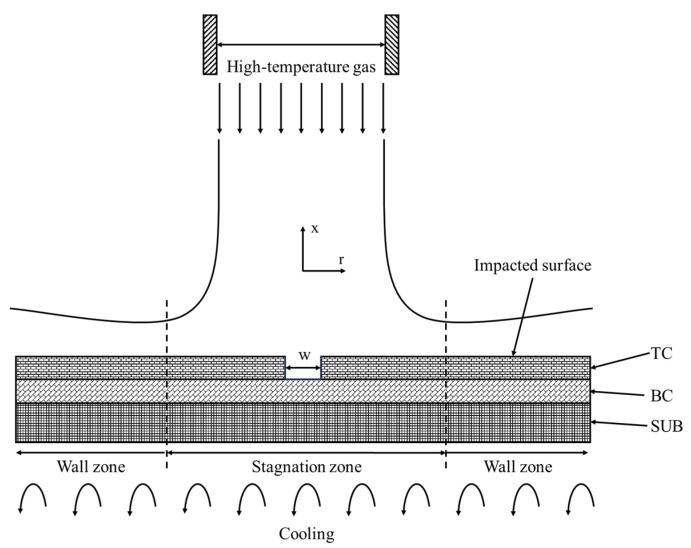
Conjugate heat transfer numerical model for localized spallation of TBCs.

A two-dimensional numerical model of the localized spallation of TBCs under high-temperature gas, consisting of the solid domain and fluid domain, was established using ANSYS DesignModeler 16.0, as depicted in Figure 6. The solid domain was a TBC with a localized spallation region, including three layers: TC, BC, and SUB. The TC layer in the spallation region was fully removed from the TC–BC interface. The localized spallation region and TC had the same depth of 300 μm, and BC and SUB had thicknesses of 200 μm and 3000 μm, respectively. The localized spallation includes eight different widths, i.e., 0 μm, 10 μm, 100 μm, 500 μm, 1 mm, 3 mm, 5 mm, and 10 mm. The length of the solid model is 10 times the total thickness of the TBCs, while the length of the fluid domain is large enough to guarantee the full flow of the high-temperature gas. The TC surface was exposed to high-temperature gas, and the back face of SUB was exposed to constant cooling air. The orientation of the inlet high-temperature gas was assumed to be perpendicular to the TC surface, and the outlet of high-temperature gas was parallel to the TC surface and flowed sideways.

The mesh of both the fluid and solid domain was generated in the ANSYS meshing. The mesh near the interface between the fluid domain and the solid domain was refined, especially. The boundary layer region of the fluid domain was refined and consisted of eight layers of O-type mesh, which ensures the dimensionless wall distance (Y+) meets the requirements of the turbulence model. The mesh nodes were well-matched at the interface between solid and fluid domains. The stretched ratio of the mesh was in the range of 0.5 to 3, which guaranteed better calculation accuracy. Approximately 240,000 high-quality meshes were generated in the two domains with an average mesh quality of 0.8. An independence and sensitivity test of the mesh was performed, with the temperature results showing a less than 1% error.

### 3.2. The Conjugate Heat Transfer Numerical Method

The temperature distribution of the localized spallation system was calculated using the CHT method, including two main processes: flow characteristics and heat transfer in the fluid domain and thermal conduction within TBCs. The high-temperature gas in the gas turbines was supposed to be inviscid and compressible, due to its high Reynolds number and light viscosity [26,27,28]. In the conjugate heat transfer process, the flow characteristics and temperature distribution were solved with heat conduction in the localized spallation regions of TBCs. Additionally, it is worth noting that the cooling condition of the SUB back face is defined by cooling temperature and convection heat transfer coefficient. At the fluid–solid interface, the continuous boundary conditions of temperature and heat flux are specified as follows [29,30,31,32,33,34]:

(1) $$T_{f}=T_{s}$$

(2) $$-k_{s}\frac{\partial T_{s}}{\partial n}=k_{f}\frac{\partial T_{f}}{\partial n}$$

where $T_{f}$ and $T_{s}$ are the temperatures of the fluid and solid domains at the coupling surface, $k_{s}$ and $k_{f}$ are the thermal conductivity of the fluid and solid domains, and n represents the normal direction.

### 3.3. Material Parameters and Boundary Conditions

The material parameters of the TBCs are summarized in Table 2 [35]. The TC, BC, and SUB are typically 8 wt% yttria-stabilized zirconia (8YSZ), NiCoCrAlY, and superalloy Inconel 718, respectively. The boundary conditions were set based on the actual operating conditions of gas turbines. The free-stream turbulence intensities and the turbulence viscosity ratios of the high-temperature gas inlet and outlet were set as 5% and 10, respectively [35]. The high-temperature gas was assumed to be an ideal gas model. The inlet of the gas was configured as the velocity boundary with an inlet temperature of 1723.15 K, a pressure of 1.9 MPa, and a uniform velocity of 130 m/s. The outlet was set as the pressure boundary with a constant pressure of 1.9 MPa. The values of cooling temperature and convective heat transfer coefficient in the cooling boundary conditions were 600 K and 600 W/(m^2^·°C), respectively, which were applied to the SUB back face. The cooling boundary conditions were designed to reduce the difference between the simulation results and the experimental results of the TC layer when the spallation width was 0 mm, which reflected the actual service conditions as much as possible. The interface between the fluid and the solid domain was set as a coupled wall. All other surfaces were assumed to be adiabatic and have non-slip velocities.

The flow characteristics and temperature distributions were synchronously resolved using the CHT procedure available in the commercial software ANSYS Fluent 16.0. The k–epsilon turbulence model and the couple algorithm were adopted to catch the flow characteristics and temperature distributions in the localized spallation region of TBCs [26]. When the residuals of each item were lower than 10^−4^, the calculation was considered to be converged. With the thicknesses of the TC, BC, and SUB and the parameters of high-temperature gas fixed, a series of calculations holding different spallation widths were performed, and the temperature distributions as well as the flow characteristics of high-temperature gas were obtained.

## 4. Results and Discussions

### 4.1. Temperature Distributions in the Localized Spallation Region

#### 4.1.1. Experimental Results

**Figure 7:**
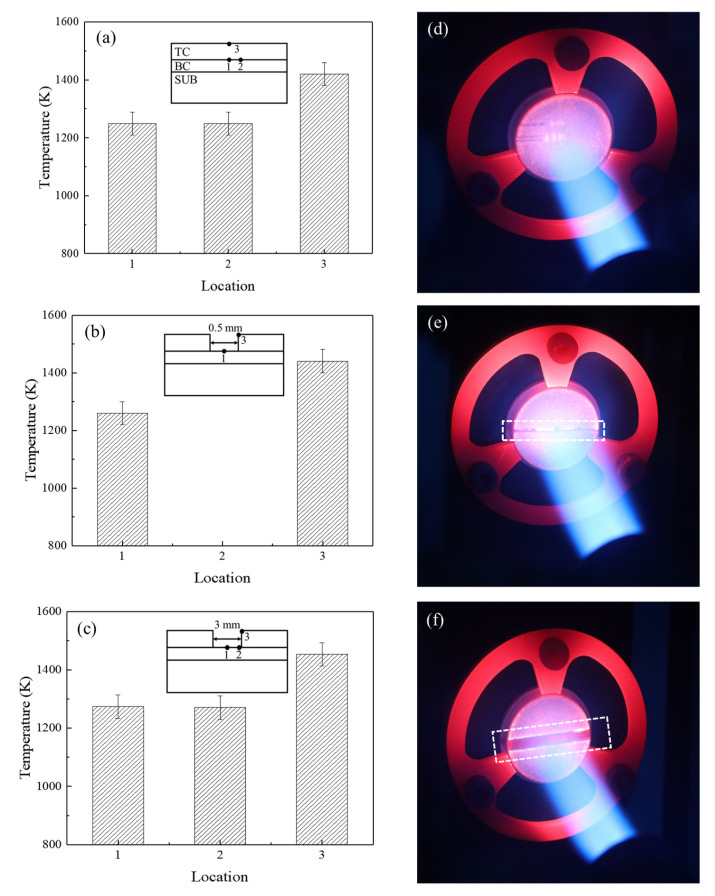
Temperature values in TBCs with spallation widths of (**a**) 0 mm, (**b**) 0.5 mm, and (**c**) 3 mm, and service statuses of TBCs with spallation widths of (**d**) 0 mm, (**e**) 0.5 mm, (**f**) 3 mm.

Figure 7 displays the measured temperature values and photographs of the TBC’s surface under a high-temperature flame. It was seen that once the spallation occurred in the TBCs, the temperatures on the TC surface and the exposed BC surface rose significantly. When the spallation width increased from 0 mm to 0.5 mm (Figure 7a,b), the temperature increase on the TC surface was approximately 20 K, which was larger than the temperature increase of about 10 K when the spallation width increased from 1 mm to 3 mm (Figure 7b,c). The edge of the spallation region was brighter than the other regions on the TC surface, indicating that the temperature in the spallation edge was higher than that in the other locations (Figure 7d–f). This was attributed to the scouring effect of the airflow, and can be considered as the over-temperature phenomenon of the localized spallation of TBCs.

Moreover, the increase in temperature on the exposed BC surface due to spallation enlargement from 0 mm to 0.5 mm was roughly 11 K (Figure 7a,b), which was lower than the temperature increase of around 14 K when the spallation region increased from 1 mm to 3 mm (Figure 7b,c). The phenomenon can be attributed to the fact that when the width of localized spallation was small, the high-temperature gas primarily came into contact with the TC layer in the spallation region, causing the temperature of the TC layer to increase rapidly. At the same time, the temperature of the BC layer experienced a slow increase. However, as the width of the spallation gradually expanded, the heat exchange between the high-temperature gas and the BC layer became more intense, resulting in a slower temperature increase rate in the TC layer and an accelerated temperature increase rate in the BC layer. Additionally, the temperature at location 1 was slightly higher than that at location 2 when the spallation increased to 3 mm. This is because when the spallation width was large enough, the high-temperature gas directly impacted the surface of the exposed BC layer, causing the temperature at the impact point to be higher than the temperature in the nearby area.

Similar features of the temperature variation are also found in other research. Previous experiments have investigated the local heat transfer coefficient of a defect on a structure when the structure is exposed to a hot mainstream [36,37]. They found that a small defect could increase the heat transfer behavior of the defect significantly. The maximum heat transfer coefficient and the temperature change occurred at the edge of the defect. Additionally, with the increase in the defect size, the heat transfer coefficient at the edge of the defect increases. These phenomena of local heat transfer coefficient are consistent with temperature variation in the experimental results.

#### 4.1.2. Numerical Results

**Figure 8:**
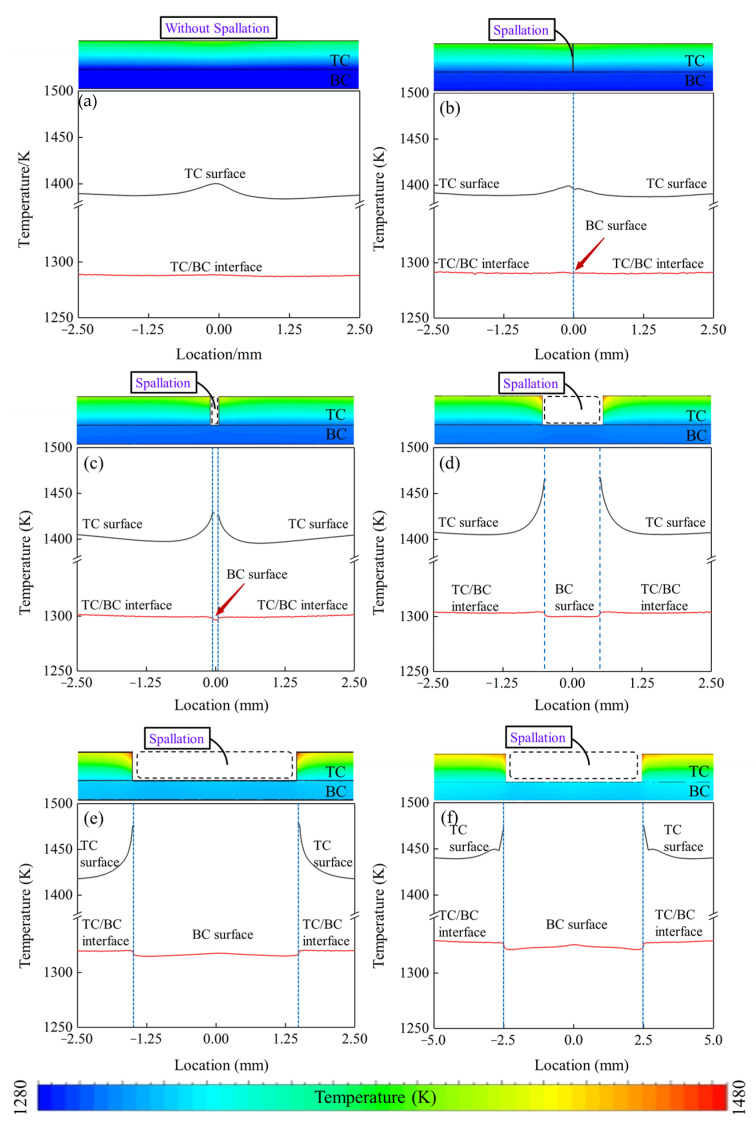
Temperature distributions in TBCs with spallation widths of (**a**) 0 μm, (**b**) 10 μm, (**c**) 100 μm, (**d**) 1000 μm, (**e**) 3000 μm, and (**f**) 5000 μm.

The temperature variations of TBCs with different spallation widths are illustrated in Figure 8, along with the corresponding temperature distributions. For the temperature along the TC surface, the results indicated that when the TBCs were intact or the spallation width was less than 10 μm (Figure 8a,b), the highest temperatures appeared in the middle of the spallation region where the values were about 1400 K, and the temperature variation along TC surface could be negligible. When the spallation reached 100 μm (Figure 8c), the overall temperature and the temperature variation dramatically increased. The highest temperatures appeared at the two corners of the spallation region on the TC surface, with values of maximum temperature and temperature variation of about 1430 K and 35 K, respectively. As the spallation further enlarged to 1 mm (Figure 8d), both the maximum temperature and temperature variation sharply rose to about 1468 K and 60 K, respectively, which may cause significant thermal mismatch stress in the TC layer. Nevertheless, when the spallation continuously expanded to 3 mm (Figure 8e), the maximum temperature only slightly rose from 1468 K to 1476 K, and the temperature variation remained nearly unchanged. With the further enlargement of the spallation, the maximum temperature was almost constant, as shown in Figure 8f, and the temperature variation reduced to about 35 K. These results suggest that expanding spallation areas can lead to a significant temperature rise in the TC layer.

For the temperature along the exposed BC surface in the localized spallation region, the results revealed that when the TBC was intact or the spallation width was less than 10 μm (Figure 8a,b), the maximum temperatures at the exposed BC surface were almost identical at 1289 K and 1292 K, and the temperature variation along the exposed BC surface could be negligible. While, when the spallation reached 100 μm (Figure 8c), a considerable increase in both the overall temperature and temperature variation along the exposed BC surface occurred, with maximum values of 1298 K and 5 K, respectively. Additionally, due to the extremely high temperature at the edge of the spallation region on the TC surface, the temperature in the TC–BC interface below the spallation edge was higher than the temperature in the exposed surface of the BC layer in the spallation region. As the spallation width enlarged to 1 mm (Figure 8d), the maximum temperature and temperature variation continued to rise to approximately 1304 K and 5 K. Once the spallation continuously expanded to 3 mm (Figure 8e), the maximum temperature at the exposed BC surface dramatically increased to 1320 K. With further enlargement of the spallation (Figure 8f), the maximum temperature and temperature variation had a lower rise, and the values were about 1326 K and 10 K, respectively.

These results suggest that expanding spallation areas can lead to a significant temperature rise in the TBCs with localized spallation. Compared to the temperature of the exposed BC layer, the temperature of the TC layer was more affected by local spallation, and the TC layer could still provide the thermal insulation function. Ekkad et al. [38] built a turbine blade leading-edge model with localized spallation, and experimentally compared the detailed heat transfer coefficients inside and outside the spallation region in a suction-type wind tunnel. They found that the heat transfer coefficient of the surface was higher than that in the spallation region, which can explain the temperature variation between the TC layer and the exposed BC layer in the numerical results. Musalek et al. [39] conducted thermal gradient tests of TBCs and observed the localized spallation in the TBCs. They found that although the BC layer in the spallation region was exposed to a high temperature and the TBC surface temperature was higher than the melting point of the BC layer, no BC layer melting was observed in the test. This phenomenon illustrates that the TBCs with spallation still retained thermal insulation function until the end of the test, which is consistent with the phenomena in numerical results.

**Figure 9:**
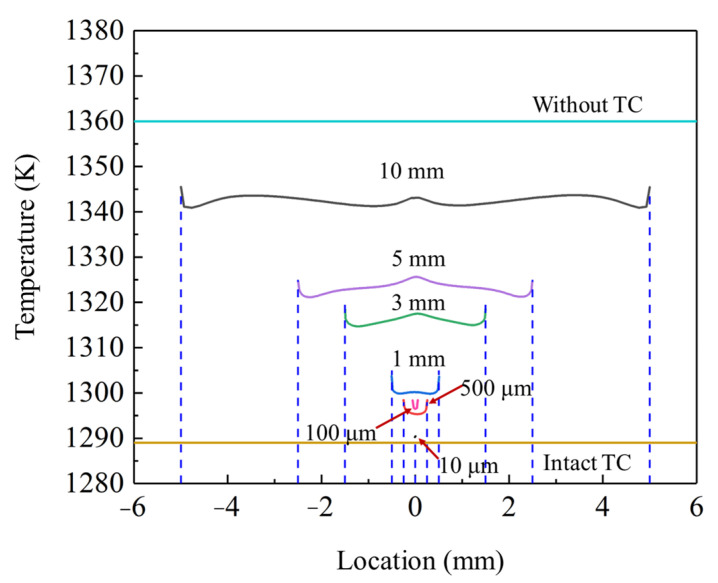
Temperature distributions on the exposed BC surface with the range of spallation widths.

Figure 9 summarizes the exposed BC surface temperature distributions with different spallation widths, considering the TBCs without TC and with intact TC as the limiting conditions. The results indicated that when the spallation width was less than 10 μm, the temperature in the localized spallation region was nearly identical to the minimum limiting value. As the spallation width increased beyond 100 μm, the overall temperature experienced a small increment. Once the spallation width exceeded 3 mm, the temperature value sharply rose and gradually approached the maximum limiting value.

**Figure 10:**
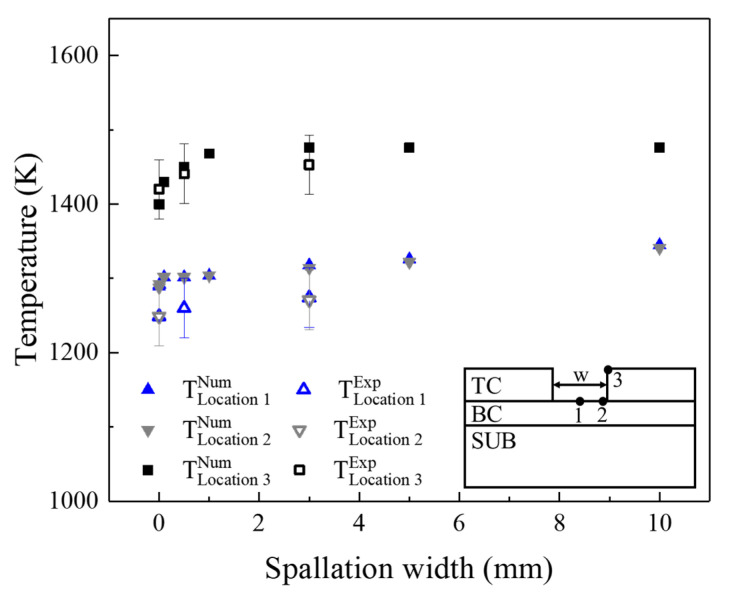
Experimental and numerical results of temperatures on the TC surface and the exposed BC surface.

To compare experimental and numerical results, the maximum temperature values at the key locations were analyzed. The experimental results, taken from the measurement points in Figure 7, were compared to the numerical data at similar positions to those in the experiments. Once the spallation occurred in TBCs, the temperature in the TC layer and BC layer increased rapidly. Meanwhile, as the spallation width increased, experiments and simulations both showed that the temperature of the TC layer first rapidly increased and then slowly increased, while the temperature of the BC layer first slowly increased and then rapidly increased. Therefore, the evolution law of the numerical results was the same as that of the experimental results, and the maximum error was within 5%, as shown in Figure 10. However, as the exposed temperature measured by the thermocouple was the average temperature within a diameter of 0.5 mm, the temperatures at location 1 and location 2 were lower than that in the simulation.

### 4.2. Flow Characteristics in the Localized Spallation Region

**Figure 11:**
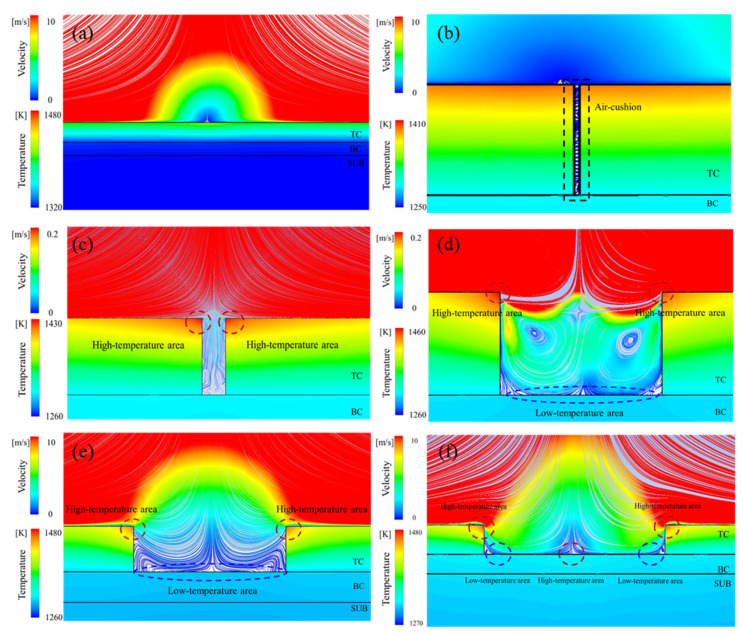
The distributions of streamline and temperature in TBCs with spallation widths of (**a**) 0 μm, (**b**) 10 μm, (**c**) 100 μm, (**d**) 500 μm, (**e**) 1000 μm, and (**f**) 3000 μm.

Based on the above results, it is worth noting that once the spallation exceeded a certain limit, the temperatures on the TC surface and the exposed BC surface rose significantly. To better understand the over-temperature phenomenon and the underlying mechanisms influenced by the localized spallation, the distributions of streamline and temperature in the spallation region were synchronously obtained and analyzed using the CFD-POST software 16.0, as illustrated in Figure 11. When the spallation was less than 10 μm (Figure 11a,b), the gas flow velocity in the spallation region was close to zero. The gas in the spallation region with extremely low speeds may be treated as an “air cushion”, which slows down the external high-temperature gas flow into the spallation region, resulting in a weak impression on the temperature field. However, as the spallation increased to 100 μm (Figure 11c), the gas flow increased to about 0~0.2 m/s in the spallation region. Although the gas velocity was still relatively low, it contributed significantly to an increase in temperature in the TBCs. In particular, the freestream gas flow reached the exposed BC surface at the spallation region and was subsequently forced to return to the gas path, accelerating the flow as it moved past the spallation edge (the two corners of the spallation region on the TC surface). This led to the highest temperature appearing at the spallation edge and significant temperature variations along the TC surface and exposed BC surface. When the spallation increased to 500 μm and even 1 mm (Figure 11d,e), an obvious backflow occurred in the spallation region, and the gas velocity near the spallation edge dramatically increased. This resulted in a significant increase in temperature along the TC surface. However, because the gas near the exposed BC surface still had a relatively low velocity, the temperature increase along the exposed BC surface was smaller than that along the TC surface. As the spallation increased to more than 3 mm (Figure 11f), the flow distributions near the spallation region gradually stabilized, and the maximum temperature value in the TC became almost constant. However, with the enlargement of the spallation width, the influence area of the gas near the spallation edge increased, which reduced the temperature variation along the TC surface. Meanwhile, the external high-temperature gas directly impacted the exposed BC surface, resulting in a significant increase in the temperature along the exposed BC surface.

Previous research has measured similar features of flow characteristics and heat transfer behavior. They monitored the flow velocity and turbulent fluctuations near the spallation region in a suction-type wind tunnel [40]. The results showed that the actual spallation size was much larger than the local boundary layer thickness of gas, and the larger spallation disturbed the boundary layer significantly. This is the key reason for the change in local heat transfer distributions. Additionally, freestream turbulence produced a higher heat transfer coefficient inside and outside the spallation. Therefore, when the spallation width was less than 10 μm, the boundary layer of the gas was not disturbed and the temperature did not change significantly. With the increase in the spallation, the freestream turbulence in the spallation region increased and led to the rapid increase in the temperature of TBCs. As the spallation width further increased, the flow velocity within the spallation area gradually stabilized, and the temperature of the local spallation region of TBCs was no longer sensitive to the spallation width.

### 4.3. Over-Temperature Damage Induced by Localized Spallation

From the previous analysis, once the TBCs with a spallation width greater than 10 μm were exposed to the high-temperature gas, complicated gas flow characteristics and temperature distribution arose in the spallation region, leading to a significant increase in temperature. As the width of the spallation increased, the TBCs lost their protective function partially, and the temperature in the affected region rose sharply. This would result in a decrease in the physical performance of the TBCs, promote TGO growth, and accelerate TBC spallation and even the cracking of the underlying blades.

To make better use of the validated numerical results to evaluate the over-temperature damage of localized spallation of TBCs, a concept of the over-temperature coefficient χ is defined, which is expressed as follows:

(3) $$\chi =\frac{T-T_{0}}{T_{0}}$$

where T is the maximum temperature on the TC surface or the exposed BC surface, and $T_{0}$ is the maximum temperature on the TC–BC interface in the intact TBCs. 

**Figure 12:**
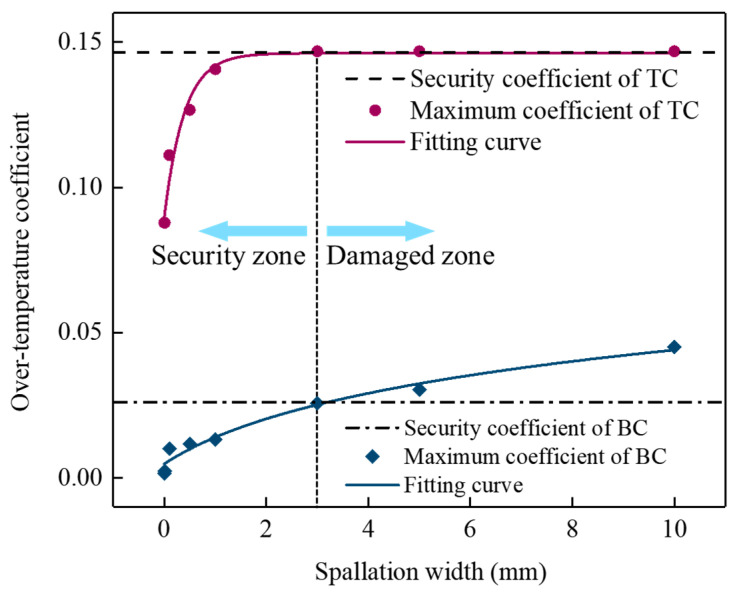
Over-temperature coefficient and nonlinear fitting results for TC and BC in TBCs with the range of spallation widths.

Based on the numerical results, the over-temperature coefficient diagram of the TBCs as a function of the spallation width was plotted in Figure 12. The statistical nonlinear fitting process was utilized to obtain the results of the nonlinear fitting curve for the over-temperature coefficient concerning spallation width, which can be used to reveal and predict the evolution of the over-temperature coefficient. A potential function was employed to describe the variation trend of the over-temperature coefficient by using initial parameter values, and the objective function was used to determine the difference between the predicted values and the actual values. The Levenberg–Marquardt algorithm was used to adjust the parameter values and minimize the objective function. The fitting process was iterated until the goodness of fit $R^{2}$ reached about 0.98.

The nonlinear fitting equation of the over-temperature coefficient in the TC layer is expressed as follows:

(4) $$\chi_{TC}=1.146-0.059e^{-\frac{w+0.015}{0.391}}$$

The nonlinear fitting equation of the over-temperature coefficient in the BC layer is expressed as follows:

(5) $$\chi_{BC}=0.993{\left(w+1.762\right)}^{0.020}$$

Considering that the sintering temperature $T_{s}$ is usually lower than the melting and the phase transition temperature, the sintering temperature may be used as a reasonable standard to judge the security of each layer [34]. The security coefficient is expressed as follows:

(6) $$\zeta =\frac{T_{s}-T_{0}}{T_{0}}$$

The sintering temperature $T_{s}$ of TC (8YSZ) was 1473 K, and that of BC (NiCoCrAlY) was 1323 K [34]. Therefore, the security coefficients ζ of the TC and BC layers were considered as 0.143 and 0.026, respectively. According to the nonlinear fitting results, the over-temperature coefficients of the TC and BC layer were simultaneously close to the security coefficient when the spallation width reached about 3 mm, as shown in Figure 12. Under this condition, the performance of TBCs degraded rapidly, which may be difficult to service safely and provide thermal protection for turbine blades. This spallation width can be considered as the critical width for the availability evaluation of localized spallation of TBCs. Here, the spallation width of the TC layer greater than 3 mm in one direction can be considered as failure, which is consistent with the failure criterion for TBCs based on experience in actual application [41].

## 5. Conclusions

In this work, the over-temperature phenomenon and internal mechanisms induced by localized spallation in TBCs were investigated through numerical and experimental methods. Conjugate heat transfer numerical implementations with different spallation widths were carried out to explore the temperature distributions and flow characteristics in the spallation region, where the temperatures at key locations are validated by thermal insulation experiments. Ultimately, a concept of the over-temperature coefficient was proposed to describe the over-temperature damage and identify the critical spallation width, which may serve as a valuable metric for evaluating the performance limits of TBCs. The main conclusions are summarized as follows:

(1) The temperature variations induced by localized spallation were given, which was validated by the numerical results. When the spallation width was less than 10 μm, the temperature in the TBCs did not change, and the TBCs were in a safe state during service. Once the spallation exceeded about 10 μm, the over-temperature phenomenon occurred in TBCs, and the temperature rose significantly. It may bring significant thermal mismatch stress in the TC layer and accelerate the non-uniform sintering and premature failure of TBCs. As the spallation increased to more than 3 mm, the maximum temperature in the TBCs became almost constant and gradually approached the security temperature, and the spallation width of 3 mm could be used as the critical spallation size for safety service, above which the TBCs fails and below which it is regarded as sustainable;
(2) The flow characteristics of the high-temperature gas were significantly affected by the localized spallation, which brought different temperature changes in the TBCs. When the spallation was less than 10 μm, the gas flow velocity in the spallation region was close to zero, which slowed down the external gas flow into the spallation region, and resulted in a weak impression on the temperature field. With the further increase in spallation width, the gas velocity in the spallation region gradually increased and brought significant temperature changes in TBCs. Once the spallation increased to more than 3 mm, the flow distributions near the spallation region gradually stabilized, and the maximum temperature value in the TBCs became almost constant;
(3) A concept of the over-temperature coefficient was proposed to identify the over-temperature damage induced by the localized spallation. The nonlinear fitting equation was obtained to reveal and predict the evolution of the over-temperature coefficient. The over-temperature coefficient may serve as a valuable metric for evaluating the performance limits of TBCs, and the results provide a foundation for the development of effective evaluation methods for surface-damaged TBCs.

## Figures and Tables

**Table 1:** Detailed thermal spray parameters for the BC layer and TC layer.

Parameters	APS for BC	APS for TC
Gun	F4MB	F4MB
Distance of gun to SUB (mm)	120	120
Current (A)	600	550
Power (kW)	42.7	42.7
Feeding rate (%)	30	25
Thickness (mm)	0.2	0.3
Gas transport (Ar) (L/min)	2.5	2.5
Primary plasma gas (Ar) (L/min)	65	35
Secondary plasma gas (H_2_) (L/min)	6.5	8

**Table 2:** Physical properties for different layers in TBCs [35].

	T (°C)	K (W/m/K)	C (J/kg/K)	ρ (kg/m^3^)
TC	-	1.05	483	5650
BC	25	4.3	501	7320
	400	6.4	592	
	800	10.2	781	
	1000	11.1	764	
SUB	100	11.4	544	8110
	300	14.9		
	500	18.3		
	700	21.8		
	900	25.2		
	1100	28.7		

## Data Availability

The original contributions presented in the study are included in the article, further inquiries can be directed to the corresponding author.
